# A computational procedure for functional characterization of potential marker genes from molecular data: Alzheimer's as a case study

**DOI:** 10.1186/1755-8794-4-55

**Published:** 2011-07-05

**Authors:** Margherita Squillario, Annalisa Barla

**Affiliations:** 1 Department of Computer and Information Science (DISI), Università degli Studi di Genova, Via Dodecaneso 35, Genova, I-16146, Italy

## Abstract

**Background:**

A molecular characterization of Alzheimer's Disease (AD) is the key to the identification of altered gene sets that lead to AD progression. We rely on the assumption that candidate marker genes for a given disease belong to specific pathogenic pathways, and we aim at unveiling those pathways stable across tissues, treatments and measurement systems. In this context, we analyzed three heterogeneous datasets, two microarray gene expression sets and one protein abundance set, applying a recently proposed feature selection method based on regularization.

**Results:**

For each dataset we identified a signature that was successively evaluated both from the computational and functional characterization viewpoints, estimating the classification error and retrieving the most relevant biological knowledge from different repositories. Each signature includes genes already known to be related to AD and genes that are likely to be involved in the pathogenesis or in the disease progression. The integrated analysis revealed a meaningful overlap at the functional level.

**Conclusions:**

The identification of three gene signatures showing a relevant overlap of pathways and ontologies, increases the likelihood of finding potential marker genes for AD.

## Background

Alzheimer's Disease (AD) is a common progressive brain disease generally diagnosed in individuals over 65 years of age and it is mostly characterized by cognition deterioration that causes dementia [1]. Within 3 to 9 years after diagnosis, it usually leads to death.

From the molecular point of view, AD is characterized by many different lesions: the most evident are deposits of beta amyloid and tangles of hyperphosphorylated tau proteins, together with a marked loss of neurons in the neocortex and hippocampus [2,3]. In the early stages, the most common symptom is memory loss, followed by mood swings, difficult in speech, long-memory loss and confusion. Several characteristics of AD are common to normal aging or to other neurological diseases, making its diagnosis very difficult. Usually, psycho-logical tests are used to indicate the presence of the disease, but only a post-mortem exam can confirm it. The diagnostic process is time-consuming and, by the time AD is detected, the disease has been progressing for many years, causing increased brain damages along with the deterioration of cognitive capacities. For these reasons, AD patients need constant care from their relatives or from specialized structures. Clearly, this phenomenon has a relevant economical impact on the national health systems.

Although many scientific papers are published every year, AD is still a very open research topic and its etiology is still unknown. In this context, the mainstream focus is to understand the underlying molecular mechanisms with the ultimate goal of identifying potential biomarkers to be used in the clinical practice.

The basis of our work is the assumption that candidate marker genes for a given disease belong to specific pathogenic pathways. Our aim was to uncover molecular pathways that are stable across tissues, treatments and measurement systems. The identification of these pathways or functional groups across different datasets is fundamental to unveil those that really feed the progression of the disease and that might harbor relevant genes.

We considered AD as a case study and obtained results from the supervised analysis of three publicly available datasets: one that collects the abundance of 120 signaling proteins [4] and two, retrieved from the Gene Expression Omnibus (GEO) database, that store gene expression data from DNA microarray experiments: GSE1297 [5] and GSE5281 [6,7]. The rationale behind [4] is very convincing and motivated our choice: since the brain controls many body functions through the release of signaling proteins in the blood stream, a brain disease like AD could induce unique changes of these proteins in the blood. We chose GSE1297 because it is homogeneous with the protein dataset for the Mini-Mental State Exam (MMSE) parameter (t-test, p-value < 0.01), which is a 30-points questionnaire test that is commonly used to screen for cognitive impairment. Unfortunately, for GSE5281 the MMSE parameter was not available, but we used it anyway because its platform, i.e. Affymetrix HG-U133 Plus2.0, provides a more accurate coverage of the human genome and completely includes the probesets measured with Affymetrix HG-U133A (GSE1297).

Supervised analysis of high-throughput data allows for the identification of lists of genes with good prediction ability. In the remainder of the paper we refer to such lists as *signatures*. Gene signature analysis is fundamental to discover the most relevant functional classes or biological pathways involved in the progression of disease.

In this work, we adopted a supervised analysis schema: *l*_1_*l*_2*FS *_regularization with double optimization framework, set in a nested cross-validation structure (*l*_1_*l*_2*FS*_). This method is inspired by [8] and it was recently proven to be robust and very effective for high-throughput data analysis [9]. The statistical accuracy of the system was measured by its prediction error that is the ability of predicting the outcome on future data (see Materials and Methods) [10].

By separately applying *l*_1_*l*_2*FS *_to each dataset, we obtained three AD signatures all showing high prediction performances. The small overlap between the two microarray signatures confirmed the necessity to consider more data coming from the same measuring technique and also different kind of data in order to incorporate all the genes that are significantly modulated by the disease.

The analysis was completed by a functional characterization of each signature in the Medical Literature Analysis and Retrieval System Online (Medline) [11], Gene Ontology (GO) [12] and the Kyoto Encyclopedia of Genes and Genomes (KEGG) [13]. This final step identified a functional overlap of ontologies and pathways. Even if the majority of the discriminant genes were different, they were frequently involved in the same KEGG pathways and/or shared similar GO ontologies. Moreover, the presence in each signature of some genes already known to be involved in the disease confirmed the reliability of the method in selecting relevant genes and also increased the likelihood that the remaining selected genes could be involved in the development of AD.

## Results

The first purpose of this work was to define significant signatures that are gene or protein lists able to distinguish, with a certain degree of reliability, diseased from control subjects. The second purpose was to test the biological soundness of the genes selected by the adopted statistical method. The third and main goal was to characterize AD at a functional level, identifying those pathways and functions that are stable across heterogeneous data sources.

*l*_1_*l*_2*FS *_is a rather novel method for feature selection and classification but it has recently been applied with success in the analysis of data coming from high-throughput techniques [14-17]. We are convinced that the ability of detecting correlated features is the most relevant property of *l_1_l_2FS_*, since correlation is a peculiar and important property characterizing the genes. It is relevant to note that, in this context, the correlation parameter *μ *in *l*_1_*l*_2*FS *_is not a threshold value, but it is a regularization parameter within the *naïve elastic net *functional (see Materials and Methods section). It allows for detecting correlated genes that contribute to the final outcome in a multivariate fashion.

Our analysis was based on one protein dataset [4] and two microarray datasets [5,6]. The obtained results are presented in this order: the classification error estimated by *l_1_l_2FS_*, a bibliographic (Medline) characterization of the relevant variables (proteins and probesets) in the signature, the results of the WebGestalt enrichment analysis performed in KEGG and GO and the analysis of the significant gene groups identified by the *k*-means clustering technique. The Medline bibliographic content we considered relevant concerns: the potential role in AD, in other brain diseases, in pathways already known to be related with AD, or the specific expression in some brain regions. Additional data on the enrichment analysis is available in the Additional files section.

### Protein data analysis

#### Results of the *l*_1_*l*_2*FS*_

**Figure 1:**
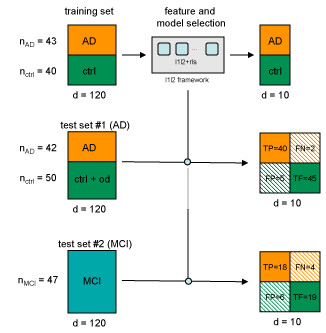
**Proteomic Training and Test (AD and MCI)**. The *Training Set *is composed by 43 AD samples and 40 NDC samples. The *Test Set AD *is composed by 42 AD samples and 50 non-AD samples (9 NDC and 11 OD). The *Test Set MCI *is composed by 47 samples diagnosed with a mild cognitive impairment and with known final follow-up (2-6 years). The *Training Set *is fed as input to the *l*_1_*l*_2*FS *_framework (grey box), which splits the data in *K *subsplits (light blue boxes), evaluating the relevant variables and the classification error for each one. Performance of the classifier is then tested on the *Test Set AD *and *Test Set MCI *using only the 21 proteins selected in the training phase. The test error is decomposed in True Positives (TP), False Negatives (FN), False Positives (FP), True Negatives (TN).

The analysis of the protein dataset consisted in two main phases. As shown in Figure 1, we firstly trained *l*_1_*l*_2*FS *_on the *Training Set*, learning a predictive statistical model and evaluating a cross-validation error. We then assessed the generalization ability of the results on independent datasets (*Test Set AD *and *Test Set MCI - Mild Cognitive Impairment*). The algorithm distinguished AD and control samples with a 10-fold cross-validation error of 19%. The presented signature corresponds to the highest value of the correlation parameter *μ *and it is composed by 21 genes, reported in Table 1. The frequency score associated to each gene indicates its stability (presence) across the lists produced by *l*_1_*l*_2*FS *_in the cross-validation procedure.

**Table 1:** Table of protein signature

Gene symbol (d)	Official Gene Symbol	Entrez ID	Frequency(%)
EGF_1	EGF	1950	100
PDGF-BB_1	PDGFB	5155	100
RANTES_1	CCL5	6352	100
TNF*_α_*	TNF*_α_*	7124	100
GCSF_1	CSF3	1440	100
ICAM-1_1	ICAM1	3383	100
IL-1*_α_*	IL-1*_α_*	3552	90
M-CSF_1	CSF1	1435	90
PARC_1	CCL18	6362	80
Acrp30_1	ADIPOQ	9370	70
ANG-2_1	ANGPT2	285	70
IL-8_1	IL8	3576	60
IL-3_1	IL3	3562	60
IL-11_1	IL11	3589	60
IL-6 R_1	IL6R	3570	50
IGFBP-6_1	IGFBP6	3489	50
MSP-a_1	MST1	4485	50
TRAIL R3_1	TNFRSF10C	8794	40
ANG_1	ANGTP1	284	40
AgRP(ART)_1	AGRP	181	40
TRAIL R4_1	TNFRSF10D	8793	40

The 21 gene signature identified by *l*_1_*l*_2*FS*_. The Gene symbol column reports the corresponding genes following the same nomenclature used by [4]. The Official Gene Symbol column shows the nomenclature of Entrez-Gene and the Frequency column shows the cross-validation frequency scores.

Following Ray et al. [4] and Ravetti and Moscato [18], we used the *Test Set AD *and the *Test Set MCI *to verify the predicting ability of our signature.

Ray et al. adopted a shrunken centroid algorithm and identified 18 predictors characterizing AD status. Similarly, Ravetti and Moscato considered the dataset and applied more than 20 different classifiers to achieve a highly predictive 5-protein signature.

After the feature selection step, for each test set the test phase consisted in extracting the sub-matrix corresponding to the 21 relevant variables identified in the training phase and in applying the learned model.

The Test Set AD is composed by samples affected by either AD or other dementia and by controls. In this case, our model scored a 7/92 error (see Figure 1), while Ray and co-authors obtained a 10/92 error and Ravetti and Moscato an error of ~ 6/92, averaged over all the methods they applied.

The *Test Set MCI *is composed by 47 samples corresponding to subjects with MCI as illustrated in Figure 1. In this case, we used the statistical model as a predictor of outcome, considering the conversion to AD as benchmark status (follow-up: 2-6 years from MCI diagnosis). The statistical model scored a 10/47 error, while Ray and co-authors obtained a 9/47 overall error and Ravetti and Moscato achieved an average error of ~ 16/47.

#### Literature characterization

Table 1 reports the 21 relevant genes identified by *l*_1_*l*_2*FS*_, ranked according their stability in terms of the frequency score. Thirteen genes are meaning-fully associated to AD, to other brain diseases or to brain-related processes. The signature completely includes the one of Ravetti and Moscato [18] and almost completely the one presented in [4]. Some genes uniquely belong to our signature: ADIPOQ, MST1, TNFRSF10C, ANGTP1, AGRP and IL6; with the exception of the latter, the other proteins have never been associated to AD. ADIPOQ encodes for the adiponectin protein that circulates in the plasma and it is involved in the metabolic and hormonal processes. This protein is unable to cross the blood-brain barrier but it is able to modify cytokine expression in the brain endothelial cells [19]; the cytokines are known to be involved in AD. ADIPOQ also characterizes the pathogenesis of the insulin resistance [20] that is a common trait of AD patients.

AGRP encodes for a protein homolog to *agouti*, a murine protein that regulates the hypothalamic control of feeding behavior via melanocortin receptor and/or intracellular calcium regulation. It therefore influences the weight homeostasis. Kim et al. [21] note that AGRP stimulates insulin secretion through calcium release in pancreatic beta cells. Imbalances in insulin and calcium are well-known risk factors for AD.

ANGPT1 and ANGPT2 contribute to the glucose metabolism by interacting with VEGF [22] and they are both indicated to have a prognostic value in adult forms of malignant brain tumors [23].

TNFR10C encodes for a member of the tumor necrosis factor that protects the cells from TRAIL-induced apoptosis. It is regulated by p53 and it is inducible by DNA damage. It is not constitutively expressed in the human brain but its apoptosis-mediating and apoptosis-blocking receptors are found on neurons, astrocytes and oligodendrocytes [24].

The same holds for TNFRSF10D, which is involved in the same KEGG pathways of TNFRSF10C (i.e. *apoptosis, cytokine-cytokine receptor interaction, natural killer cell mediated cytotoxicity*).

MST1 encodes for the macrophage stimulating 1 factor. MST1, interacting with FOXO1, induces its accumulation in the nucleus leading to cell death, upon withdrawal of growth factors and neuronal activity [25].

#### Functional analysis of the signature

Table 2 shows the results of gene set enrichment analysis of the signature using KEGG [26].

**Table 2:** Table of the functional analysis made in KEGG for protein signature

KEGG pathway	KEGG category	**n**.	P
**Cytokine-cytokine receptor interaction**		14	9.69e-26
Cell adhesion molecules (CAMs)	Signaling Molecules and Interaction	1	7.59e-2
Neuroactive ligand-receptor interaction		1	1.67e-1

**Hematopoietic cell lineage**		7	6.00e-14
**Natural killer cell mediated cytotoxicity**		4	9.12e-7
**Toll-like receptor signaling pathway**	Immune System	3	2.19e-5
Fc epsilon RI signaling pathway		2	1.01e-3
Leukocyte transendothelial migration		2	2.29e-3
T cell receptor signaling pathway		1	5.57e-2

**Apoptosis**	Cell Growth and Death	5	1.54e-9

**MAPK signaling pathway**		4	2.21e-5
**Jak-STAT signaling pathway**	Signal Transduction	4	1.90e-6
TGF-beta signaling pathway		1	4.96e-2

**Adipocytokine signaling pathway**	Endocrine System	3	1.13e-5
PPAR signaling pathway		1	4.17e-2

Epithelial cell signaling in Helicobacter pylori infection	Infectious Diseases	2	8.05e-4

Gap junction	Cell Communication	2	1.33e-3
Focal adhesion		2	6.43e-3

Glioma	Cancers	2	6.66e-4
Pancreatic cancer		1	4.42e-2

Type I diabetes mellitus	Metabolic Diseases	2	2.96e-4
Type II diabetes mellitus		2	3.26e-4

Regulation of actin cytoskeleton	Cell Motility	2	6.89e-3

Enriched pathways of the protein signature resulting from the WebGestalt functional analysis with KEGG. For each pathway, the table reports the pathway name, its KEGG category, the number of selected genes (n.) and the p-value. The enriched pathways are displayed in boldface.

**Figure 2:**
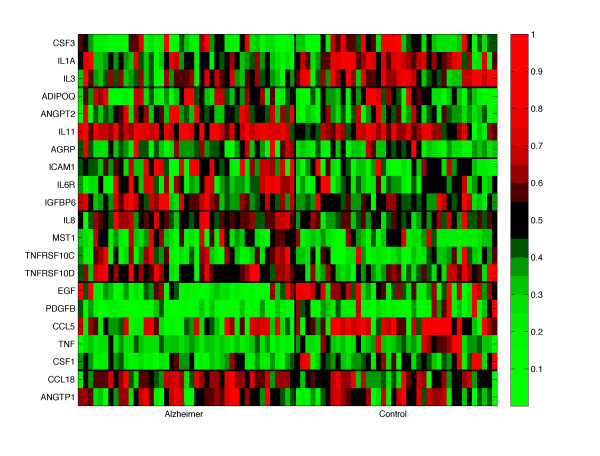
**Heatmap for the proteomic signature**. Heatmap representing the abundance of the 21 selected proteins by *l*_1_*l*_2*FS*_. The thick black lines show the gene groups identified with the k-means clustering technique. The samples are divided in two classes: AD and control. The red and green colors represent high and low abundance respectively. For visualization purposes the expressions have been scaled between 0 and 1.

The selected proteins are especially involved in the Signaling Molecules and Interaction and Immune System categories, but also in processes related to the cell (Cell Growth and Death, Cell Communication, Signal Transduction). These results underline the role of the selected genes within pathways already linked to AD: *cytokine-cytokine receptor interaction *[2,3,27], *hematopoietic cell lineage *[2,3], *apoptosis *[2,3,27], pathways involved in the immune and inflammatory response [2] and pathways related to Metabolic Diseases [28]. We also identified pathways not previously associated with AD: *adipocytokine, PPAR signaling pathway, glioma *and *pancreatic cancer*. We extended the functional analysis of our signature applying the gene set enrichment procedure on GO. The results are presented in Additional file 1. The heatmap plot in Figure 2 visualizes the structured signature obtained by *l*_1_*l*_2*FS *_and postprocessed by *k*-means clustering. Such structured representation confirmed that the genes belonging to the same clusters, having highly correlated abundance profiles, are indeed grouped in the same ontologies or biological pathways or they are known to interact. For instance, gene set enrichment in GO showed two gene pairs: GCSF/IL3 in the positive biological processes and IL8/TNFRSF10D in the negative biological processes. The heatmap plot in Figure 2 shows them into two different clusters. The enrichment in KEGG provided additional examples. For instance, EGF and PDGFB were clustered together and they are both involved in several pathways: *cytokine-cytokine receptor interaction, MAPK signaling pathway, gap junction, focal adhesion, glioma and regulation of actin cytoskeleton*. TNF*_α _*and CSF1 show similar abundance profiles and they are included in the *hematopoietic cell lineage and cytokine-cytokine signaling *pathways. These proteins are also known to interact. Similar examples are: IL3/IL-1*_α_*, CSF3/IL-1*_α_*, ADIPOQ/AGRP, TNFRSF10C/TNFRS10D.

### Microarray data analysis - GSE1297

#### Results of the *l_1_l_2FS_*

The *l_1_l_2FS _*procedure applied to the GSE1297 dataset provided a signature of 12 probesets associated to 11 genes, reported in Table 3. The list corresponds to the highest value of the correlation parameter *μ*. In this case the 10-fold cross-validation error was 17%. Nine of the 11 genes are related to AD, other neurological disorders or brain functions. It is not straightforward to compare our results to those in [5], because they applied a Pearson correlation test, without providing a classification error.

**Table 3:** Table of gene expression signature (GSE1297)

Gene symbol (d)	Official Gene Symbol	Entrez ID	Frequency(%)
221728_x_at	XIST	7503	100
221729_at	COL5A2	1290	100
221730_at	COL5A2	1290	100
206552_s_at	TAC1	6863	90
200800_s_at	HSPA1A	3303	60
200664_s_at	DNAJB1	3337	50
201645_at	TNC	3371	50
204337_at	RGS4	5999	50
212063_at	CD44	960	50
213436_at	CNR1	1268	50
202018_s_at	LTF	4057	40
220122_at	MCTP1	79772	40

The 12 probesets signature identified by *l*_1_*l*_2*FS*_. The Gene symbol refers to the Affymetrix probeset ID, the Official Gene Symbol shows the nomenclature of Entrez-Gene and the Frequency column shows the cross-validation frequency score.

#### Literature characterization

XIST encodes for a transcript not translated in a protein. Its main role is the X chromosome inactivation during the early development in mammal females. More recently, one transcription variant of XIST has been found expressed in a subset of neurons as part of a group of gender-specific genes differentially expressed in some brain regions [29].

The literature describes TAC1 as the encoder for many hormones that may function as neurotransmitters, interacting with nerve receptors and smooth muscle cells.

HSPA1A is a heat shock protein of the Hsp70 family. It prevents the subsequent aggregation of misfolded proteins and it is instrumental in targeting them for degradation when the above mechanism fails [30]. The misfolding and the aggregation of proteins are common characteristics of several neurodegenerative diseases including AD and Parkinson's disease, therefore it is very likely that HSPA1A may also characterize AD.

Heat shock protein DNAJB1 has an increased expression in the lymphoblastoid cell lines from patients with bipolar I and II disorders and schizophrenia [31]. It is also known to modulate the activity of the Hsp70 family that leads to the translocation of proteins into the mitochondria and endoplasmatic reticulum thus affecting the functions of these organelles [32].

TNC encodes tenascin-C that contributes to the invasive nature of glioblastoma and the majority of high-grade gliomas [33], to the adhesion of medulloblastoma [34] and to the malignant transformation of plexiform neurofibromas [35].

RGS4 belongs to the regulators of G protein signaling family. A recent paper [36] reports that RGS4 contributes to the regional differences in the coupling of muscarinic M1 receptors in AD. The expression of this gene in the human brain might be spatially and temporally regulated by alternative promoters, resulting in several previously unknown isoforms [37]. This may have implications for the physiological role of RGS4 in some pathologies of the brain.

Besides being involved in the cannabinoid-induced central nervous system (CNS) effects, cannabinoid receptor 1 (CNR1) participates in the development of insulin resistance in the human skeletal muscle and its expression is markedly decreased in AD brains [38].

LTF is a major iron-binding protein in milk and body secretions with an antimicrobial activity. It has several functions, including regulation of iron homeostasis, host defense against a broad range of microbial infections, anti-inflammatory activity, regulation of cellular growth and differentiation. This protein seems to be up-regulated in AD patients [39].

MCTP1 encodes for a protein integral to the cell membrane that is involved in the calcium-mediated signaling process. Indeed, the imbalance of calcium is one of the major AD risk factors.

#### Functional analysis of the signature

Table 4 and Additional file 2 present the results of the functional characterization performed in KEGG and GO.

**Table 4:** Table of the functional analysis made in KEGG for GSE1297 signature

KEGG pathway	KEGG Category	**n**.	P
**ECM-receptor interaction**	Signaling Molecules and Interaction	3	2.39e-6
Neuroactive ligand-receptor interaction		2	3.93e-3

Cell Communication	Cell Communication	2	5.40e-4
Focal adhesion		2	1.75e-3

MAPK signaling pathway	Signal Transduction	1	8.51e-2

Antigen processing and presentation	Immune System	1	2.37e-2
Hematopoietic cell lineage		1	2.76e-2

Enriched pathways of the GSE1297 signature resulting from the WebGestalt functional analysis with KEGG. For each pathway, the table reports the pathway name, its KEGG category, the number of selected genes (n.) and the p-value. The enriched pathways are displayed in boldface.

KEGG analysis indicated that these genes are especially involved in the Signaling Molecules and Interaction KEGG category (*ECM-receptor-interaction and neuroactive ligand-receptor interaction*)and in Cell Communication and Immune System (*cell communication, focal adhesion, antigen processing and presentation and hematopoietic cell lineage*).

**Figure 3:**
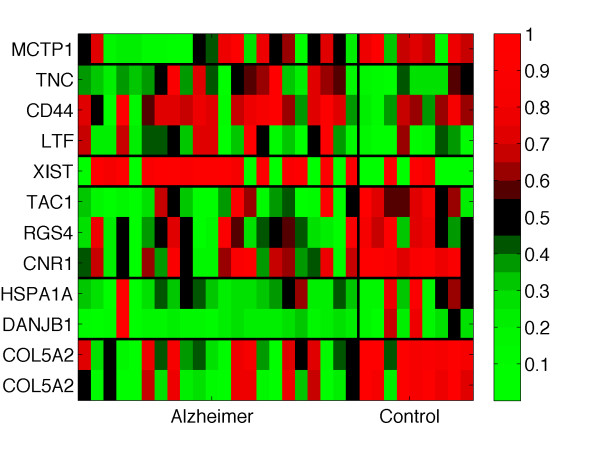
**Heatmap for the GSE1297 signature**. Heatmap representing the gene expression of the 12 selected probestes by *l*_1_*l*_2*FS *_in the GSE1297 experiment. The thick black lines show the gene groups identified with the k-means clustering technique. The samples are divided in two classes: AD and control. The red and green colors represent high and low expression respectively. For visualization purposes the expressions have been scaled between 0 and 1.

Again, we considered the functional results together with the structured heatmap, identifying those groups of probesets with similar expression profiles also involved in the same pathways or in the same ontologies, see Figure 3. For instance, CNR1, TAC1 and RGS4 are involved in the *G-protein coupled receptor protein signaling pathway *GO process; CNR1 and TAC1 are involved in the *neuroactive ligand-receptor interaction *KEGG pathway. CD44 and TNC share the ECM-*receptor interaction *pathway. The two probesets referring to the same gene, COL5A2, were nicely clustered together in the same group and showed very similar expression profiles. The last pair is composed by HSPA1 and DNAJB1 which have similar expression profiles and belong to the same biological process, i.e. *response to stimulus*. These proteins are also known to interact [40].

### Microarray data analysis - GSE5281

#### Results of the *l_1_l_2FS_*

Table 5 reports the 39 probesets that formed the second microarray-based signature, associated with the highest value of the correlation parameter *μ*. In this case, the 5-fold cross-validation error was 5%. Nine of the 39 identified genes are already associated to AD, other brain diseases or brain-related processes. As opposed to the other datasets, the number of well-characterized genes was low compared to the number of selected genes. A comparison of the classification results was not feasible because in [6] the authors used a t-test filtering method, which is not directly associated to a classification error.

**Table 5:** Table of gene expression signature (GSE5281)

Gene symbol (d)	Official Gene Symbol	Entrez ID	Frequency(%)
202234_s_at	SLC16A1	6566	100
205048_s_at	PSPH	5723	100
212063_at	CD44	960	100
213921_at	SST	6750	100
223380_s_at	LATS2	26524	100
230629_s_at	EP400	57634	100
231735_s_at	MALAT1	378938	100
235987_at	PRKXP1	441733	100
204142_at	ENOSF1	55556	80
212417_at	SCAMP1	9522	80
213872_at	C6orf62	81688	80
214246_x_at	MINK1	50488	80
217028_at	CXCR4	7852	80
220122_at	MCTP1	79772	80
220182_at	SLC25A23	79085	80
221646_s_at	ZDHHC11	79844	80
227413_at	UBLCP1	134510	80
228697_at	HINT3	135114	80
209116_x_at	HBB	3043	60
212451_at	SECISBP2L	9728	60
216834_at	RGS1	5996	60
224588_at	XIST	7503	60
228946_at	INTU	27152	60
229120_s_at	CDC42SE1	56882	60
230748_at	SLC16A6	9120	60
1554447_at	LOC554203	554203	60
1569110_x_at	LOC728613	728613	60
202436_s_at	CYP1B1	1545	40
203540_at	GFAP	2670	40
204338_s_at	RGS4	5999	40
206826_at	PMP2	5375	40
211959_at	IGFBP5	3488	40
213274_s_at	CTSB	1508	40
213791_at	PENK	5179	40
214980_at	UBE3A	7337	40
227062_at	NEAT1	283131	40
229676_at	MTPAP	55149	40
229793_at	ASAH2B	653308	40
235060_at	LOC100190986	100190986	40

The 39 probesets signature identified by *l*_1_*l*_2*FS*_. The Gene symbol refers to the Affymetrix probeset ID, the Official Gene Symbol shows the nomenclature of Entrez-Gene and the Frequency column shows the cross-validation frequency score.

#### Literature characterization

The somatostatin hormone (SST) is expressed in the body and a ects the rates of neurotransmission [41].

CXCR4, the chemokine receptor 4, is specific for stromal cell-derived factor-1. This protein is known to be highly expressed in the neural precursor cells [42], to be implicated in the inflammation affecting the brain [43] and, like several others chemokines and their receptors, to be implicated in AD [44]. This gene is also critical to the progression of various brain malignancies [45,46].

GFAP, the glial fibrillary acidic protein, encodes one of the major intermediate filament proteins of mature astrocytes. Besides its use as a marker to distinguish astrocytes from other glial cells during development [47,48], GFAP is expressed in the neurons of hippocampus of AD patients [49-51].

CTSB, the lysosomal cysteine proteinase, is known as an amyloid precursor protein (APP) secretase and it is involved in the proteolytic processing of APP. Indeed, the incomplete proteolytic processing of APP is the most known and important causative factor in AD [52].

Enkephalin precursor PENK is a neuropeptide hormone that, together with protachykinin A the precursor of SP, is altered in both dementia and acute neuroinflammatory disorders [53].

Finally, the ubiquintin protein ligase E3A (UBE3A) can be implicated not only in the pathogenesis of Angelman syndrome but also in the neurodegenerative disorders involving protein aggregation [54].

#### Functional analysis of the signature

Table 6 reports the results of the enrichment analysis in KEGG. We did not find any enriched pathway because the majority of the selected genes are not yet functionally characterized. Nevertheless, some of the involved pathways had a significant p-value (p-value < 0.05) and, among them, those in Neurodegenerative Diseases, Signaling Molecules and Interaction and Immune System are the most significantly connected with AD. The results of GO enrichment analysis are reported in Additional file 3.

**Table 6:** Table of the functional analysis made in KEGG for GSE5281 signature

KEGG pathway	KEGG Category	**n**.	P
Glycine, serine and thereonine	Amino Acid Metabolism	1	3.20e-2
Tryptophan metabolism		1	5.88e-2

Metabolism of xenobiotics by cytochrome P450	Xenobiotics Biodegradation and Metabolism	1	4.23e-2

Neurodegenerative Diseases	Neurodegenerative Diseases	1	2.54e-2
Prion disease		1	9.04e-3

MAPK signaling pathway		1	1.83e-1
Cytokine-cytokine receptor interaction		1	1.68e-1
ECM-receptor interaction	Signaling Molecules and Interaction	1	6.02e-2
Neuroactive ligand-receptor migration		1	1.96e-1

Axon guidance	Development	1	8.77e-2

Antigen processing and presentation		1	5.31e-2
Hematopoietic cell lineage	Immune System	1	6.17e-2
Leukocyte transendothelial migration		1	8.14e-2

Enriched pathways of the GSE5281 signature resulting from the WebGestalt functional analysis with KEGG. For each pathway, the table reports the pathway name, its KEGG category, the number of selected genes (n.) and the p-value. The enriched pathways are displayed in boldface.

### Integrated functional analysis of the three signatures

First, we proceeded by simply intersecting the three gene signatures. This procedure identified RGS4, MCTP1, CD44 and XIST as common between the two microarray signatures.

Taking a step towards an integrated functional characterization, we compared our results at the functional level, in order to identify common pathways and/or ontologies. The integration followed a late integration schema [55].

**Figure 4:**
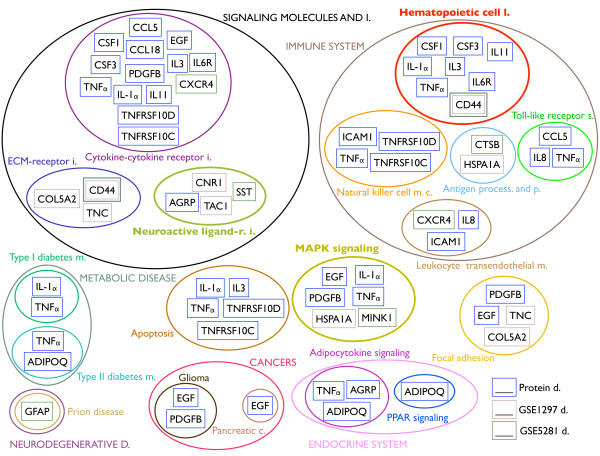
**Integrated functional analysis for the three signatures**. Relevant KEGG pathways, including: the enriched pathways, those common between two or more signatures and also those related to AD or other brain diseases (in boldface the pathways common to all the signatures). Within each pathway we report the selected genes color-coded according to the signature: purple for the protein dataset, pink for the GSE1297 dataset and dark green for GSE5281 dataset. KEGG categories are displayed in uppercase.

The comparison at the pathway level for the KEGG results is shown in Table 7 and Figure 4. Table 7 presents three common pathways and several pathways common between two of the three signatures. Figure 4 illustrates the selected genes and their relation to the aforementioned pathways. This figure summarizes the main result of the integrated analysis, highlighting the common pathways between two signatures, the pathways shared by all signatures and also those known to be related to AD or other brain diseases. The shared pathways were: *hematopietic cell lineage, neuroactive ligand-receptor interaction *and *MAPK signaling pathways*.

**Table 7:** Table of the integrated functional analysis for the three signatures

KEGG pathway	KEGG Category	Protein	GSE1297	GSE5281
Cytokine-cytokine receptor interaction		●		o
Neuroactive ligand-receptor migration	Signaling Molecules and Interaction	o	o	o
ECM-receptor interaction			●	o

Antigen processing and presentation			o	o
Hematopoietic cell lineage	Immune System	●	o	o
Leukocyte transendothelial migration		o		o

MAPK signaling pathway	Signal Transduction	●	o	o

Focal adhesion	Cell Communication	o	o	o

KEGG pathways common between two or three signatures. The table reports the pathway name, its KEGG category and the signatures IDs. The enriched pathways are identified with filled circles, whereas empty circles are associated to not enriched pathways.

In the GO integrated analysis (see Additional files) it is clear that the annotation for the protein dataset is richer than the other two datasets, hence the overlap takes place at a somewhat high level in the GO hierarchy. For instance, in the BP domain, the overlap reaches down to the *cellular process *node. If we consider two signatures at a time, we note slightly more specific common nodes. In both the protein and GSE1297 signatures, the following ontologies are enriched: *extracellular region *and *extracellular region part *in the CC domain and *response to stimulus and response to biotic stimulus *for the BP domain. If we consider the protein and GSE5281 signatures, the common enriched nodes are *negative regulation of biological process *and *negative regulation of cellular process*.

## Discussions

In this section we will clarify the reasons behind the choice of the datasets and we will illustrate more extensively the results in the context of identifying common functional pathways possibly harboring marker genes.

We primarily chose to study heterogeneous datasets to deal with the multifactorial nature of AD. Indeed, AD affects different brain areas [7] with various lesions: the most evident are deposits of beta amyloid and tangles of hyperphosphorylated tau proteins, together with a marked loss of neurons mostly in the neocortex and hippocampus [2,3]. Moreover, AD impacts many molecular levels causing the depletion of neurotrophins and neurotransmitters, dysfunctions affecting the mitochondria, disturbances affecting the metabolism of cholesterol and insulin, inflammation and loss of calcium regulation [3]. Hence, we analyzed data measuring the effects of AD at different molecular levels, i.e. DNA and proteins.

The protein dataset mainly measures the abundance of a specific class of signaling proteins while the two microarray datasets quantify the expression of nearly the entire genome. Nevertheless, we deemed the integrated functional analysis feasible because there exists an overlapping set of measured genes across the three datasets. This assumption was also validated *a posteriori*, because the number of common pathways between the microarray datasets was comparable with the number of common pathways between the protein dataset and each microarray set (see Table 7). Despite the blood protein data are heavily shifted towards cytokine measurements, the overall results were unbiased.

The integration of results from different datasets corresponds to an *in silico *validation phase. A consistency assessment of the results across datasets is fundamental in verifying their reliability and in deciding for a further biological validation step [56].

The integrated functional analysis led to very promising results: most of the significant identified genes and pathways are likely related to AD and worth further investigation.

The classification performances of the protein dataset were equivalent or slightly better than those achieved by [4], who provided the dataset, and by [18], who later analyzed it. The *l*_1_*l*_2*FS *_protein signature was comparable with those in [4,18], completely including the latter and differing on only three genes with the former. Seven genes were uniquely selected by *l*_1_*l*_2*FS*_, namely: IL6R, MST1, TNFRSF10C, ANGTP1, ADIPOQ and AGRP. Aside for IL6R, they have never been associated to AD.

Both ADIPOQ and AGRP are involved in the same KEGG pathway of TNF*_α_*, which is a protein considered to be a probable prognostic factor of AD and recently mentioned in several works [2,4,18]. This pathway, namely *adipocytokine signaling*, also comprises PGC-1*_α_*, a protein that is a potential target for treating type II diabetes (it is indeed involved in the regulation of glucose metabolism) and that has been shown to decrease the hyperglycemic-mediated production of beta-amyloid [57]. In addition, the work of Gavrila et al. [58] suggested the use of AGRP as useful peripheral marker of metabolism change. ANGPT1 as well as ANGPT2 encodes for angiopoietins, relevant for vascular development, angiogenesis and lymphogenesis. Both proteins were studied in patients with type II diabetes mellitus and found to be related to VEGF, glycemic control, endothelial damage/dysfunction and atherosclerosis [22]. In particular, ANGPT1 has a role in the pathological vascularization of malignant astrocytomas [59] and the balance between ANGPT1 and ANGPT2 has prognostic value in patients with primary glioblastoma multiforme [23]. The involvement of these proteins in the glucose metabolism as well as in the pathogenesis of brain tumors makes them interesting for further investigation with *in vitro *techniques.

The functional analysis revealed pathways previously unrelated to AD, e.g. *adipocytokine, PPAR signaling pathway *and other related to different diseases, such as *glioma *and *pancreatic cancer*. Despite having a significant p-value, these three pathways were not enriched, because of the small number of genes belonging to the signature, see Table 2.

The analysis of GSE1297 led to the identification of TAC1. TAC1 encodes for substance P (SP), neurokinin A, neuropeptide K and neuropeptide gamma. SP stimulates human peripheral blood monocytes to produce in ammatory cytokines including IL12, IL6, IL1 and TNF*_α_*, all belonging to the protein signature except for the former two. Recently, [53] highlighted the alteration of the highly correlated PENK and SP in both dementia and acute neuroinflammatory disorders. SP is a neuropeptide that is widely distributed in the central and peripheral nervous systems and it has a well-established role as neurotransmitter and as neuroimmunoregulator [60]. SP and its receptor NK1R are involved in inflammation and neurological disorders within the CNS [61,62]. PENK also belonged to the GSE5281 signature. At the functional level, the analysis selected the *ECM-receptor-interaction *and the *neuroactive ligand-receptor interaction *pathways, both belonging to the Signaling Molecules and Interaction KEGG category, which is related to AD [4].

In the GSE5281 signature, SST and UBE3A are relevant for further studies. SST is an important regulator of the endocrine system and it is likely to have a role in the regulation of ADIPOQ, AGRP and TNF*_α_*, that *l*_1_*l*_2*FS *_selected in the protein signature and that were assigned to enriched KEGG pathways within the *Endocrine system *category. SST also affects the rates of neurotransmission in the CNS and proliferation of both normal and tumorigenic cells. Associations of this gene with AD are already suggested [63-66], even if its exact role in the disease is not clear yet. SST is also known to be co-transported with SP, one of the products of TAC1 mentioned above.

UBE3A functions as a cellular quality control ubiquitin ligase. Since AD is characterized by the accumulation of amyloid-beta and tau peptides, this protein could be a good candidate for investigation because it participates to the ubiquitin protein degradation system, with the main function of breaking down the non-functional proteins.

The *l*_1_*l*_2*FS *_analysis performed separately on the microarray datasets scored low cross-validation errors and revealed 4 common significant genes: RGS4, MCTP1, CD44 and XIST. Besides RGS4 [36], the other genes have never been associated with AD but, as noted in literature characterization, they are very likely to play a role in the disease.

The integrated functional analysis showed pathways that are common across two or three of the signatures, see Table 7 and Figure 4. Such pathways are already related to AD and they belong to the *Signaling Molecules and Interaction, Immune System, Signal Transduction *and *Cell Communication *KEGG categories. In particular, these pathways are involved in the immune system and inflammation (*cytokine-cytokine receptor interaction, antigen processing and presentation, hematopoietic cell lineage, leukocyte transendothelial migration*), in the nervous system and related diseases (*neurodegenerative disorders, neuroactive ligand-receptor interaction, prion disease *and *axon guidance*), in cell proliferation, differentiation and migration and also in the maintenance of the structure and function of one cell line or tissue (*MAPK signaling and ECM-receptor interaction*).

The combined analysis of the three signatures led to the identification of common probesets between the two microarray signatures and of a meaningful functional overlap among the three signatures. The statistical method adopted for the analysis allowed for the selection of genes that are already known to be related with AD or to be expressed in the brain. This strengthened the hypothesis that the remaining selected genes might be relevant for further studies, especially because the results were obtained without using any prior knowledge on the subject. Despite the very convincing results, we are aware of some limitations that affected our work and that should be overcome in the future. From the data viewpoint, we coarsely divided the available samples in two broad classes (healthy and diseased) but it would be more accurate to separately consider the three AD stages (incipient, moderate, severe) versus controls. More-over, when dealing with the GSE5281 data, we could discard those samples extracted from the visual cortex that seems to be spared by AD [6]. On the computational side, the *l*_1_*l*_2*FS *_framework has the advantage of being correlation-aware and multivariate. Unfortunately, the algorithm itself and the double loop of nested cross-validation procedures heavily demand computational power, in contrast to more standard techniques, such as t-test, that are easier to run even by non-specialists. Nonetheless, the computational burden can be significantly reduced by running the statistical framework on distributed computing facilities, such as clusters, grids or cloud computing.

## Conclusion

Gene signatures are the first indispensable step towards the identification of genes and proteins highly related with disease and belonging to pathogenic pathways. Indeed, in the context of AD, there is a urgent need to improve its molecular characterization to establish novel therapeutic targets and reliable biomarkers.

By applying *l*_1_*l*_2*FS *_on three AD datasets, we defined gene signatures with good discriminative properties for the classification of diseased and healthy subjects. Our work, focused on the functional characterization of potentially meaningful AD genes, revealed 21 genes in the protein dataset [4], 11 and 39 genes in the GSE1297 [5] and GSE5281 [6] microarray datasets respectively. Each signature was robust from the statistical viewpoint and it was associated to a low cross-validation error. Some of the selected genes are already known to be involved in AD; others have never been associated with the disease but the current biological knowledge suggests their possible correlation with AD. Specifically, the first group comprises TNF*_α_*, IL6R, IL-1*_α_*, GFAP, CXCR4, CTSB, SST, LTF, CNR1, RGS4 and the second is formed by ADIPOQ, AGRP, HSPA1A, PENK, UBE3A, TAC1, TNFRSF10C and ANGPT1.

The functional analysis of the signatures confirmed the validity of the results, identifying pathways that are biologically meaningful and related with AD, see Figure 4.

The integrated functional analysis revealed three overlapping pathways: *hematopoietic cell lineage, neuroactive ligand-receptor interaction *and *MAPK signaling pathways*. The validity of our procedure was also confirmed at the functional level, in fact the common pathways are already mentioned in the literature as important for AD [2,3].

The use of *l*_1_*l*_2*FS *_as core algorithm for feature selection allowed for the identification of relevant and correlated genes. Therefore those genes not included in common pathways and still unmentioned in the literature could play a role in the pathogenesis of the disease.

In the near future, we plan to extend the analysis in order to include more available domain knowledge. We are currently working on designing methods that explicitly use prior knowledge on the subject, to drive the feature selection step for example adopting filtering techniques [15] or designing appropriate kernel functions [56,67]. We also plan to integrate heterogeneous data from different techniques (Chip-on-Chip, SNP, GWAS and miRNA) and possibly from different-omics domains.

## Materials and methods

### 5.1 Data

The protein dataset of Ray and co-authors [4] is a collection of 259 plasma samples from individuals with presymptomatic to late-stage AD and from various controls. The abundance of 120 well-known signaling proteins was measured for each sample with the filtered-based arrayed sandwich ELISA. The data-set is available online, already normalized and subdivided in four groups: the *Training Set*, composed by 43 AD samples and 40 nondemented control (NDC) samples; the *Test Set AD*, composed by 42 AD, 39 NDC and 11 other dementia (OD) samples; the *Test Set MCI*, composed by 47 MCI samples with follow-up (2-6 years from MCI diagnosis); the *OND and RA patient set*, composed by 21 other neurological disease (OND) and 16 rheumatoid arthrithis (RA) samples. The latter dataset was excluded from our analysis because it did not contain any AD data. The remaning test sets that presented more than two classes were considered as binary problems: AD versus not-AD.

The other datasets are stored in the GEO repository: GSE1297 of Blalock et al. [5] and GSE5281 of Liang et al. [6]. The first consisted of 22 AD samples and 9 controls retrieved from the hippocampal brain region. The gene expressions were measured on the Affymetrix HG-U133A platform. The second dataset was measured on the Affymetrix HG-U133 Plus2.0 platform and it was composed by 87 AD late onset samples and 74 controls on six different brain regions originating from the same subject: entorhinal cortex, hippocampus, medial temporal gyrus, posterior cingulate, superior frontal gyrus and primary visual cortex. Some of the samples originated from the same subject, but we did not take explicitly into account this information. The statistical framework treats those samples as if they belonged to different subjects. For both microarray datasets, the gene expressions were extracted from the. CEL files and normalized using the Robust Multichip Average method [68] by running an R [69] script, based on the *aroma package.affymetrix *[70] http://www.aroma-project.org.

### Feature selection framework

*l*_1_*l*_2*FS *_is a regularization method capable to select subsets of discriminative genes. The algorithm can be tuned to give a minimal set of discriminative genes or larger sets including correlated genes. The method is based on the optimization principle presented in [8] and further developed and studied in [9,71]. First we fix some notation and then we explain the idea behind the algorithm. Assume we are given a collection of *n *subjects, each represented by a *d*-dimensional vector *x *of measurements (e.g. the gene expression or the protein abundance vector). Each sample is also associated with a binary label *y*, assigning it to a class (e.g. AD or control). The dataset is therefore represented by a *n *× *d *matrix *X*, where *d *>>*n *and *Y *is the *n*-dimensional labels vector. The *l*_1_*l*_2*FS *_with double optimization algorithm looks for a linear model *f*(*x*) = *βx*, whose sign gives the classification rule that can be used to associate a new sample to one of the two classes. The offset is zero, since we normalize the X matrix to have zero mean. Note that the vector of weights *β *is forced to be a sparse vector, that is some of its entries are zero, then some variables (probesets or proteins) will not contribute in building the estimator *f*(*x*). The weight vector *β *is found by minimizing the *naïve elastic net *functional:

where the least square error is penalized with the *l*_1 _and *l*_2 _norm of the coefficient vector *β*. The least square term ensures fitting of the data whereas adding the two penalties allows to avoid over-fitting. The role of the two penalties is different, the *l*_1 _term (sum of absolute values) enforces the solution to be sparse, the *l*_2 _term (sum of the squares) preserves correlation among the variables. The solution of this step, computed through the simple iterative softthresholding [72], is followed by a second optimization, namely regularized least squares (RLS):

to estimate the classifier on the selected features and to improve the classification performance [73]. The training for selection and classification requires the choice of the regularization parameters for both *l*_1_*l*_2*FS *_regularization and RLS denoted with τ* and λ*, respectively. In fact, the model selection and statistical significance assessment are performed within two nested *K*-fold cross-validation loops, similarly to [14,74]. When a separate set of new data, i.e. a *test set*, is difficult to gather because of cost or time issues, cross-validation represents the most common solution to achieve an accurate estimate of prediction error. In *K*-fold cross-validation, the available data are split into *K *roughly equal-sized parts. For each *k*-th part, a model is trained on the other *K *- 1 (training) and a prediction error is evaluated using the fitted model to predict the outcome on the *k*-th part (test). The estimated prediction error is the combination (usually the average) of the *K *estimated errors. Specifically, we emploied *stratified *cross-validation, where the folds are selected so that the mean response value is approximately equal in all the folds. In the case of binary classification, this means that each fold contains roughly the same proportions of the two types of class labels. Stratified cross-validation is particularly useful to avoid unbalances between Type I (False Positive - FP) and Type II (False Negative - FN) errors.

Being interested in a comprehensive list of relevant variables, we fixed our attention on the lists obtained with the highest values for the correlation parameter *μ *(*μ *= 1). The framework was implemented in Python, based on the L1L2Py library http://slipguru.disi.unige.it/Research/L1L2Py.

The statistical framework described above provides a set of *K *lists of selected variables, therefore it it is necessary to choose an appropriate criterion [75] in order to assess a common list of relevant variables (probesets or proteins, in our case). We based ours on the absolute frequency, i.e. we decided to promote as relevant variables the most stable probesets across the lists. The threshold we used to select the final lists was chosen according to the slope variation of the number of selected genes vs. frequency (plot not shown), its value being 40%. In this way we managed to cut out those variables that were not stable across the cross-validation lists, similarly to the procedure adopted in [14].

We also visualized the signatures in heatmap plots in order to devise the modules of correlated variables within the signatures by applying a *k*-means clustering procedure, with the aim of enhancing the genes with correlating expressions [76]. We used the *correlation distance *and set the number of clusters according to the dimensionality of the minimal list that is the list selected by *l*_1_*l*_2*FS *_when the correlation parameter is set to the minimum value of *μ*.

### Functional Analysis

For the functional analysis of the signatures we used the online gene set analysis toolkit WebGestalt [77] http://bioinfo.vanderbilt.edu/webgestalt/.

This online toolkit performs the gene set enrichment in KEGG and GO and identifies the most relevant pathways and ontologies in the signatures. GO is a database of controlled vocabularies (ontologies) that describes gene products in terms of their associated domains, that are biological process (BP), cellular component (CC) and molecular function (MF), in a species independent manner. GO is structured as a directed acyclic graph where each term has a defined relationship to one or more terms in the same domain and sometimes to other domains. The most common visual representation of GO is a graph where the relations among the ontologies (*nodes*) are represented by connecting lines (*arcs*). KEGG is a repository that stores the higher-order systemic behaviors of the cell and the organism from genomic and molecular information. It is an integrated database resource consisting of 16 main databases, broadly categorized into systems information, genomic information, and chemical information. All the available KEGG pathways have been biologically validated before publishing. Both for KEGG and GO, we selected the WebGestalt human genome as reference set, p-value ≤ 0.05 as level of significance, 3 as the minimum number of genes and the default Hypergeometric test as statistical method. Medline [11] was used to retrieve the available prior knowledge on the genes from the current literature.

## List of Abbreviations

AD: Alzheimer's Disease; GEO: Gene Expression Omnibus; MMSE: Mini-Mental State Exam; *l*_1_*l*_2*FS*_*: l*_1_*l*_2*FS *_regularization with double optimization feature selection; Medline: Medical Literature Analysis and Retrieval System Online; GO: Gene Ontology; KEGG: Kyoto Encyclopedia of Genes and Genomes; MCI: Mild Cognitive Impairment; CNS: Central Nervous System; NDC: Non Demented Controls; OD: Other Dementia; OND: Other Neurological Disease; RA: Rheumathoid Arthritis; RLS: Regularized Least Squares FP: False Positive; FN: False Negative; BP: Biological Process; CC: Cellular Component; MF: Molecular Function.

## Competing interests

The authors declare that they have no competing interests.

## Authors' contributions

MS contributed to the functional analysis, the biological interpretation of the results and the writing of the paper. AB curated the data normalization and analysis and the visualization of the results. All authors read and approved the final manuscript.

## Pre-publication history

The pre-publication history for this paper can be accessed here:

http://www.biomedcentral.com/1755-8794/4/55/prepub

## Supplementary Material

Additional file 1 **Ontologies for the protein signature**. Results of WebGestalt analysis of the gene set enrichment made for the 21 protein signature in GO. The enriched ontologies are marked in red color. The most significant ontologies in MF are associated to the selected cytokines: some have chemokines activity, some belong to the hematopoietin/interferon class, some have a growth factor activity and others are coupled with the G-proteins. The most enriched process in the BP domain is cell communication, that is connected to signal transduction. More general processes follow, like regulation of cellular processes, of physiological processes and of cellular physiological processes. It is interesting to observe that these general biological processes present both positive and negative regulation, having only IL3 as common gene. IL3 is a potent growth promoting cytokine involved in several activities like cell growth, differentiation and apoptosis. IL3 possesses neurotrophic activity and it is associated with neurological disorders like schizophrenia. Click here for file

Additional file 2 **Ontologies for the GSE1297 signature**. Results of WebGestalt analysis of the gene set enrichment made for the GSE1297 signature in GO. The enriched ontologies are marked in red color. The GO enrichment is not associated to a large subgraph, as in the protein signature analysis, because not all the genes are functionally characterized. The CC domain shows that the gene products of this signature are mainly located in the *extracellular region*, probably because they are involved in the *response to stimulus *process, that it is subsequently propagated inside the cell trough the *G-protein coupled receptor protein signaling pathway*. Click here for file

Additional file 3 **Ontologies for the GSE5281 signature**. Results of WebGestalt analysis of the gene set enrichment made for the GSE5281 signature in GO. The enriched ontologies are marked in red color. Similarly to the GSE1297 analysis, the GO subgraph associated to this signature is not large because of the scarce functionally characterizion of its genes. Click here for file

## References

[B1] Gandhi S Wood NW Genome-wide association studies: the key to unlocking neurodegeneration? Nat Neurosci 2010 13 7 789 94 10.1038/nn.2584 20581814

[B2] Wyss-Coray T Inammation in Alzheimer disease: driving force, bystander or beneficial response? Nat Med 2006 12 9 1005 15 16960575 10.1038/nm1484

[B3] Querfurth HW LaFerla FM Alzheimer's disease N Engl J Med 2010 362 4 329 44 10.1056/NEJMra0909142 20107219

[B4] Ray S Britschgi M Herbert C Takeda-Uchimura Y Boxer A Blennow K Friedman L Galasko D Jutel M Karydas A Classification and prediction of clinical Alzheimer's diagnosis based on plasma signaling proteins Nat Med 2007 13 11 1359 1362 10.1038/nm1653 17934472

[B5] Blalock EM Geddes JW Chen KC Porter NM Markesbery WR Landfield PW Incipient Alzheimer's disease: microarray correlation analyses reveal major transcriptional and tumor suppressor responses Proc Natl Acad Sci USA 2004 101 7 2173 8 10.1073/pnas.0308512100 14769913 PMC357071

[B6] Liang WS Dunckley T Beach TG Grover A Mastroeni D Walker DG Caselli RJ Kukull WA McKeel D Morris JC Hulette C Schmechel D Alexander GE Reiman EM Rogers J Stephan DA Gene expression profiles in anatomically and functionally distinct regions of the normal aged human brain Physiological Genomics 2007 28 3 311 22 17077275 10.1152/physiolgenomics.00208.2006 PMC2259385

[B7] Liang WS Reiman EM Valla J Dunckley T Beach TG Grover A Niedzielko TL Schneider LE Mastroeni D Caselli RJ Kukull W Morris JC Hulette CM Schmechel D Rogers J Stephan DA Alzheimer's disease is associated with reduced expression of energy metabolism genes in posterior cingulate neurons PNAS 2008 105 11 4441 6 10.1073/pnas.0709259105 18332434 PMC2393743

[B8] Zou H Hastie T Regularization and variable selection via the elastic net Journal of the Royal Statistical Society, Series B 2005

[B9] De Mol C Mosci S Traskine M Verri A A Regularized Method for Selecting Nested Groups of Relevant Genes from Microarray Data Journal of Computational Biology 2009 16 1 15 10.1089/cmb.2008.0137 19432538

[B10] Efron B Tibshirani R An introduction to the bootstrap 1993 436

[B11] Medline http://www.ncbi.nlm.nih.gov/pubmed/

[B12] The Gene Ontology database http://www.geneontology.org http://www.geneontology.org

[B13] KEGG PATHWAY Database http://www.genome.ad.jp/kegg/pathway.html

[B14] Fardin P Barla A Mosci S Rosasco L Verri A Varesio L The l1-l2 regularization framework unmasks the hypoxia signature hidden in the transcriptome of a set of heterogeneous neuroblastoma cell lines BMC Genomics 2009 10 474 10.1186/1471-2164-10-474 19832978 PMC2768750

[B15] Fardin P Cornero A Barla A Mosci S Acquaviva M Rosasco L Gambini C Verri A Varesio L Identification of multiple hypoxia signatures in neuroblastoma cell lines by l1-l2 regularization and data reduction Journal of Biomedicine and Biotechnology 2010 10.1155/2010/878709 PMC2905945 20652058

[B16] Fardin P Barla A Mosci S Rosasco L Verri A Versteeg R Caron HN Molenaar JJ Ora I Eva A Puppo M Varesio L A biology-driven approach identifies the hypoxia gene signature as a predictor of the outcome of neuroblastoma patients Molecular Cancer 2010 10.1186/1476-4598-9-185 PMC2908582 20624283

[B17] Guzzetta G Jurman G Furlanello C A machine learning pipeline for quantitative phenotype prediction from genotype data BMC Bioinformatics 2010 10.1186/1471-2105-11-S8-S3 PMC2966290 21034428

[B18] Ravetti MG Moscato P Identification of a 5-protein biomarker molecular signature for predicting Alzheimer's disease PLoS ONE 2008 3 9 e3111 10.1371/journal.pone.0003111 18769539 PMC2518833

[B19] Spranger J Verma S Göhring I Bobbert T Seifert J Sindler AL Pfeiffer A Hileman SM Tschöp M Banks WA Adiponectin does not cross the blood-brain barrier but modifies cytokine expression of brain endothelial cells Diabetes 2006 55 141 7 10.2337/diabetes.55.01.06.db05-1077 16380487

[B20] Yang H yuan Li Y qiang Nie Y jian Zhou Y lei DU Y hong Sha W Hong Y The relationship between insulin resistance and adiponectin gene expression in nonalcoholic fatty liver disease Zhonghua Gan Zang Bing Za Zhi 2007 15 7 525 8 17669243

[B21] Kim HS Yumkham S Lee HY Cho JH Kim MH Koh DS Ryu SH Suh PG C-terminal part of AgRP stimulates insulin secretion through calcium release in pancreatic beta Rin5mf cells Neuropeptides 2005 39 4 385 93 10.1016/j.npep.2005.04.005 15978665

[B22] Lim HS Lip GYH Blann AD Angiopoietin-1 and angiopoietin-2 in diabetes mellitus: relationship to VEGF, glycaemic control, endothelial damage/dysfunction and atherosclerosis Atherosclerosis 2005 180 113 8 10.1016/j.atherosclerosis.2004.11.004 15823283

[B23] Sie M Wagemakers M Molema G Mooij JJA de Bont ESJM den Dunnen WFA The angiopoietin 1/angiopoietin 2 balance as a prognostic marker in primary glioblastoma multiforme J Neurosurg 2009 110 147 55 10.3171/2008.6.17612 18991494

[B24] Dörr J Bechmann I Waiczies S Aktas O Walczak H Krammer PH Nitsch R Zipp F Lack of tumor necrosis factor-related apoptosis-inducing ligand but presence of its receptors in the human brain J Neurosci 2002 22 4 RC209 11844843 10.1523/JNEUROSCI.22-04-j0001.2002 PMC6757573

[B25] Yuan Z Lehtinen MK Merlo P Villén J Gygi S Bonni A Regulation of neuronal cell death by MST1FOXO1 signaling J Biol Chem 2009 284 17 11285 92 19221179 10.1074/jbc.M900461200 PMC2670133

[B26] Kanehisa M Goto S Furumichi M Tanabe M Hirakawa M KEGG for representation and analysis of molecular networks involving diseases and drugs Nucleic Acids Res 2010 38 Database D355 60 10.1093/nar/gkp896 PMC2808910 19880382

[B27] Colangelo V Schurr J Ball MJ Pelaez RP Bazan NG Lukiw WJ Gene expression profiling of 12633 genes in Alzheimer hippocampal CA1: transcription and neurotrophic factor down-regulation and up-regulation of apoptotic and pro-inammatory signaling J Neurosci Res 2002 70 3 462 73 10.1002/jnr.10351 12391607

[B28] Kroner Z The relationship between Alzheimer's disease and diabetes: Type 3 diabetes? Altern Med Rev 2009 14 4 373 9 20030463

[B29] Vawter MP Evans S Choudary P Tomita H Meador-Woodruff J Molnar M Li J Lopez JF Myers R Cox D Watson SJ Akil H Jones EG Bunney WE Gender-specific gene expression in post-mortem human brain: localization to sex chromosomes Neuropsy-chopharmacology 2004 29 2 373 84 10.1038/sj.npp.1300337 PMC3130534 14583743

[B30] Luk KC Mills IP Trojanowski JQ Lee VMY Interactions between Hsp70 and the hydrophobic core of alpha-synuclein inhibit fibril assembly Biochemistry 2008 47 47 12614 25 10.1021/bi801475r 18975920 PMC2648307

[B31] Iwamoto K Bundo M Washizuka S Kakiuchi C Kato T Expression of HSPF1 and LIM in the lymphoblastoid cells derived from patients with bipolar disorder and schizophrenia J Hum Genet 2004 49 5 227 31 10.1007/s10038-004-0136-5 15362566

[B32] Iwamoto K Kakiuchi C Bundo M Ikeda K Kato T Molecular characterization of bipolar disorder by comparing gene expression profiles of postmortem brains of major mental disorders Mol Psychiatry 2004 9 4 406 16 10.1038/sj.mp.4001437 14743183

[B33] Hirata E Arakawa Y Shirahata M Yamaguchi M Kishi Y Okada T Takahashi JA Matsuda M Hashimoto N Endogenous tenascin-C enhances glioblastoma invasion with reactive change of surrounding brain tissue Cancer Science 2009 100 8 1451 9 10.1111/j.1349-7006.2009.01189.x 19459858 PMC11158953

[B34] Wegmann D Dupanloup I Excoffier L Width of gene expression profile drives alternative splicing PLoS ONE 2008 3 10 e3587 10.1371/journal.pone.0003587 18974852 PMC2575406

[B35] Lévy P Ripoche H Laurendeau I Lazar V Ortonne N Parfait B Leroy K Wechsler J Salmon I Wolkenstein P Dessen P Vidaud M Vidaud D Bièche I Microarray-based identification of tenascin C and tenascin XB, genes possibly involved in tumorigenesis associated with neurofibromatosis type 1 Clin Cancer Res 2007 13 2 Pt 1 398 407 17202312 10.1158/1078-0432.CCR-06-0182

[B36] Muma NA Mariyappa R Williams K Lee JM Differences in regional and subcellular localization of G(q/11) and RGS4 protein levels in Alzheimer's disease: correlation with muscarinic M1 receptor binding parameters Synapse 2003 47 58 65 10.1002/syn.10153 12422374

[B37] Ding L Mychaleckyj JC Hegde AN Full length cloning and expression analysis of splice variants of regulator of G-protein signaling RGS4 in human and murine brain Gene 2007 401 1-2 46 60 10.1016/j.gene.2007.07.002 17707117

[B38] Ramírez BG Blázquez C del Pulgar TG Guzmán M de Ceballos ML Prevention of Alzheimer's disease pathology by cannabinoids: neuroprotection mediated by blockade of microglial activation J Neurosci 2005 25 8 1904 13 10.1523/JNEUROSCI.4540-04.2005 15728830 PMC6726060

[B39] An L Sato H Konishi Y Walker DG Beach TG Rogers J Tooyama I Expression and localization of lactotransferrin messenger RNA in the cortex of Alzheimer's disease Neurosci Lett 2009 452 3 277 80 10.1016/j.neulet.2009.01.071 19348738 PMC2667624

[B40] Michels AA Kanon B Bensaude O Kampinga HH Heat shock protein (Hsp) 40 mutants inhibit Hsp70 in mammalian cells J Biol Chem 1999 274 51 36757 63 10.1074/jbc.274.51.36757 10593983

[B41] Samson WK Zhang JV Avsian-Kretchmer O Cui K Yosten GLC Klein C Lyu RM Wang YX Chen XQ Yang J Price CJ Hoyda TD Ferguson AV bin Yuan X Chang JK Hsueh AJW Neuronostatin encoded by the somatostatin gene regulates neuronal, cardiovascular, and metabolic functions J Biol Chem 2008 283 46 31949 59 10.1074/jbc.M804784200 18753129 PMC2581552

[B42] Ni HT Hu S Sheng WS Olson JM Cheeran MCJ Chan ASH Lokensgard JR Peterson PK High-level expression of functional chemokine receptor CXCR4 on human neural precursor cells Brain Res Dev Brain Res 2004 152 2 159 69 15351504 10.1016/j.devbrainres.2004.06.015

[B43] Chvapil M Peng YM Oxygen and lung fibrosis Arch Environ Health 1975 30 11 528 32 1217912 10.1080/00039896.1975.10666770

[B44] Weeraratna AT Kalehua A Deleon I Bertak D Maher G Wade MS Lustig A Becker KG Wood W Walker DG Beach TG Taub DD Alterations in immunological and neurological gene expression patterns in Alzheimer's disease tissues Exp Cell Res 2007 313 3 450 61 10.1016/j.yexcr.2006.10.028 17188679 PMC2565515

[B45] Barbero S Bajetto A Bonavia R Porcile C Piccioli P Pirani P Ravetti JL Zona G Spaziante R Florio T Schettini G Expression of the chemokine receptor CXCR4 and its ligand stromal cell-derived factor 1 in human brain tumors and their involvement in glial proliferation in vitro Ann N Y Acad Sci 2002 973 60 9 10.1111/j.1749-6632.2002.tb04607.x 12485835

[B46] Rubin JB Kung AL Klein RS Chan JA Sun Y Schmidt K Kieran MW Luster AD Segal RA A small-molecule antagonist of CXCR4 inhibits intracranial growth of primary brain tumors PNAS 2003 100 23 13513 8 10.1073/pnas.2235846100 14595012 PMC263845

[B47] Mouser PE Head E Ha KH Rohn TT Caspasemediated cleavage of glial fibrillary acidic protein within degenerating astrocytes of the Alzheimer's disease brain Am J Pathol 2006 168 3 936 46 10.2353/ajpath.2006.050798 16507909 PMC1606516

[B48] Wharton SB O'Callaghan JP Savva GM Nicoll JAR Matthews F Simpson JE Forster G Shaw PJ Brayne C Ince PG Function MC Group ANS Population variation in glial fibrillary acidic protein levels in brain ageing: relationship to Alzheimer-type pathology and dementia Dement Geriatr Cogn Disord 2009 27 5 465 73 10.1159/000217729 19420941

[B49] Korolainen MA Auriola S Nyman TA Alafuzoff I Pirttilä T Proteomic analysis of glial fibrillary acidic protein in Alzheimer's disease and aging brain Neurobiol Dis 2005 20 3 858 70 10.1016/j.nbd.2005.05.021 15979880

[B50] Hol EM Roelofs RF Moraal E Sonnemans MAF Sluijs JA Proper EA de Graan PNE Fischer DF van Leeuwen FW Neuronal expression of GFAP in patients with Alzheimer pathology and identification of novel GFAP splice forms Mol Psychiatry 2003 8 9 786 96 10.1038/sj.mp.4001379 12931206

[B51] Middeldorp J van den Berge SA Aronica E Speijer D Hol EM Specific human astrocyte subtype revealed by affinity purified GFAP antibody; unpurified serum cross-reacts with neurofilament-L in Alzheimer PLoS ONE 2009 4 11 e7663 10.1371/journal.pone.0007663 19888461 PMC2766629

[B52] Tagawa K Kunishita T Maruyama K Yoshikawa K Kominami E Tsuchiya T Suzuki K Tabira T Sugita H Ishiura S Alzheimer's disease amyloid beta-clipping enzyme (APP secretase): identification, purification, and characterization of the enzyme Biochem Biophys Res Commun 1991 177 377 87 10.1016/0006-291X(91)91994-N 1645961

[B53] Ernst A Buerger K Hartmann O Dodel R Noelker C Sommer N Schwarz M Köhrle J Bergmann A Hampel H Midregional Proenkephalin A and N-terminal Protachykinin A are decreased in the cerebrospinal fluid of patients with dementia disorders and acute neuroinammation J Neuroimmunol 2010 221 1-2 62 7 10.1016/j.jneuroim.2010.02.004 20207019

[B54] Mishra A Dikshit P Purkayastha S Sharma J Nukina N Jana NR E6-AP promotes misfolded polyglutamine proteins for proteasomal degradation and suppresses polyglutamine protein aggregation and toxicity J Biol Chem 2008 283 12 7648 56 10.1074/jbc.M706620200 18201976

[B55] Pavlidis P Weston J Cai J Noble Grundy W Gene functional classification from heterogeneous data Proceedings of the fifth annual international conference on Computational biology 2001

[B56] Hamid JS Hu P Roslin NM Ling V Greenwood CMT Beyene J Data Integration in Genetics and Genomics: Methods and Challenges Human Genomics and Proteomics 2009 2009 1 13 10.4061/2009/869093 PMC2950414 20948564

[B57] Qin W Haroutunian V Katsel P Cardozo CP Ho L Buxbaum JD Pasinetti GM PGC-1alpha expression decreases in the Alzheimer disease brain as a function of dementia Arch Neurol 2009 66 3 352 61 10.1001/archneurol.2008.588 19273754 PMC3052997

[B58] Gavrila A Chan JL Miller LC Heist K Yiannakouris N Mantzoros CS Circulating melanin-concentrating hormone, agouti-related protein, and alpha-melanocyte-stimulating hormone levels in relation to body composition: alterations in response to food deprivation and recombinant human leptin administration J Clin Endocrinol Metab 2005 90 2 1047 54 15546902 10.1210/jc.2004-1124

[B59] Zadeh G Reti R Koushan K Baoping Q Shannon P Guha A Regulation of the pathological vasculature of malignant astrocytomas by angiopoietin-1 Neoplasia (New York, NY) 2005 7 12 1081 90 10.1593/neo.05424 PMC1501179 16354591

[B60] Li Y Douglas SD Pleasure DE Lai J Guo C Bannerman P Williams M Ho W Human neuronal cells (NT2-N) express functional substance P and neurokinin-1 receptor coupled to MIP-1 beta expression J Neurosci Res 2003 71 4 559 66 10.1002/jnr.10504 12548712 PMC4015112

[B61] Mantyh PW Johnson DJ Boehmer CG Catton MD Vinters HV Maggio JE Too HP Vigna SR Substance P receptor binding sites are expressed by glia in vivo after neuronal injury PNAS 1989 86 13 5193 7 10.1073/pnas.86.13.5193 2472640 PMC297584

[B62] Kostyk SK Kowall NW Hauser SL Substance P immunoreactive astrocytes are present in multiple sclerosis plaques Brain Res 1989 504 2 284 8 10.1016/0006-8993(89)91369-3 2480834

[B63] Saiz-Sanchez D Ubeda-Bañon I de la Rosa-Prieto C Argandoña-Palacios L Garcia-Muñozguren S Insausti R Martinez-Marcos A Somatostatin, tau, and beta-amyloid within the anterior olfactory nucleus in Alzheimer disease Exp Neurol 2010 223 2 347 50 10.1016/j.expneurol.2009.06.010 19559700

[B64] Xue S Jia L Jia J Association between somatostatin gene polymorphisms and sporadic Alzheimer's disease in Chinese population Neurosci Lett 2009 465 2 181 3 10.1016/j.neulet.2009.09.002 19733630

[B65] Vepsäläinen S Helisalmi S Koivisto AM Tapaninen T Hiltunen M Soininen H Somatostatin genetic variants modify the risk for Alzheimer's disease among Finnish patients J Neurol 2007 254 11 1504 8 10.1007/s00415-007-0539-2 17987251

[B66] van de Nes JAP Konermann S Nafe R Swaab DF Beta-protein/A4 deposits are not associated with hyperphosphorylated tau in somatostatin neurons in the hypothalamus of Alzheimer's disease patients Acta Neuropathol 2006 111 2 126 38 10.1007/s00401-005-0018-8 16456666

[B67] Lanckriet GRG Bie TD Cristianini N Jordan MI Noble WS A statistical framework for genomic data fusion Bioinformatics 2004 20 16 2626 35 10.1093/bioinformatics/bth294 15130933

[B68] Irizarry R Bolstad B Collin F Cope L Hobbs B Speed T Summaries of Affymetrix GeneChip probe level data Nucleic Acids Res 2003 31 4 e15 10.1093/nar/gng015 12582260 PMC150247

[B69] Team RDC R: A language and environment for statistical computing R Foundation for Statistical Computing 2004

[B70] Hansen K Bullard J Simpson K Bengtsson H aroma.affymetrix: A generic framework in R for analyzing small to very large Affymetrix data sets in bunded memory Tech Report #745 Department of Statistics, University of California, Berkeley, February 2008

[B71] De Mol C De Vito E Rosasco L Elastic Net Regularization in Learning Theory Journal of Complexity 2009

[B72] Daubechies I Defrise M De Mol C An iterative thresholding algorithm for linear inverse problems with a sparsity constraint arXiv 2003 math.FA

[B73] Candes E Tao T The Dantzig selector: statistical estimation when p is much larger than n Annals of Statistics 2007

[B74] Barla A Mosci S Rosasco L Verri A A method for robust variable selection with significance assessment Proceedings of ESANN 2008 2008

[B75] Jurman G Merler S Barla A Paoli S Galea A Furlanello C Algebraic stability indicators for ranked lists in molecular profiling Bioinformatics 2008 24 2 258 264 10.1093/bioinformatics/btm550 18024475

[B76] Mosci S Barla A Verri A Rosasco L Finding structured gene signatures IEEE Proceedings BIBM 2008 2008 8

[B77] Zhang B Kirov S Snoddy J WebGestalt: an integrated system for exploring gene sets in various biological contexts Nucleic Acids Res 2005 33 Web Server W741 8 10.1093/nar/gki475 PMC1160236 15980575

