# Supplementary figures and images for: A computational procedure for functional characterization of potential marker genes from molecular data: Alzheimer's as a case study
Source: BMC Med Genomics. 2011 Jul 5;4:55. doi: 10.1186/1755-8794-4-55 (PMC3149568; doi:10.1186/1755-8794-4-55)

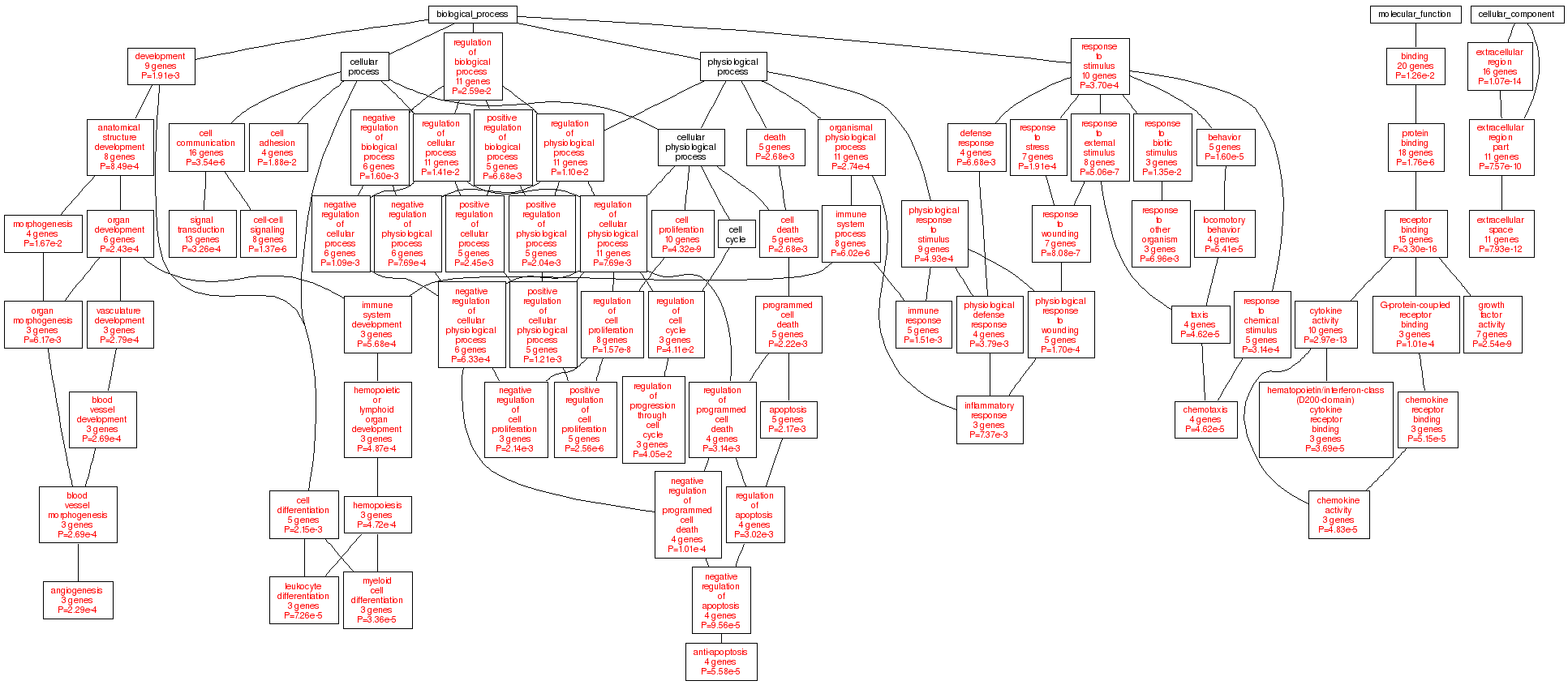

Supplement: Additional file 1 — Ontologies for the protein signature. Results of WebGestalt analysis of the gene set enrichment made for the 21 protein signature in GO. The enriched ontologies are marked in red color. The most significant ontologies in MF are associated to the selected cytokines: some have chemokines activity, some belong to the hematopoietin/interferon class, some have a growth factor activity and others are coupled with the G-proteins. The most enriched process in the BP domain is cell communication, that is connected to signal transduction. More general processes follow, like regulation of cellular processes, of physiological processes and of cellular physiological processes. It is interesting to observe that these general biological processes present both positive and negative regulation, having only IL3 as common gene. IL3 is a potent growth promoting cytokine involved in several activities like cell growth, differentiation and apoptosis. IL3 possesses neurotrophic activity and it is associated with neurological disorders like schizophrenia. [file 1755-8794-4-55-S1.PNG]

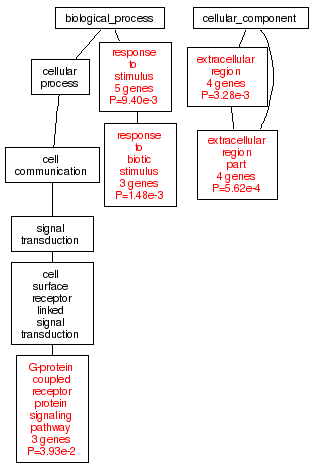

Supplement: Additional file 2 — Ontologies for the GSE1297 signature. Results of WebGestalt analysis of the gene set enrichment made for the GSE1297 signature in GO. The enriched ontologies are marked in red color. The GO enrichment is not associated to a large subgraph, as in the protein signature analysis, because not all the genes are functionally characterized. The CC domain shows that the gene products of this signature are mainly located in the extracellular region, probably because they are involved in the response to stimulus process, that it is subsequently propagated inside the cell trough the G-protein coupled receptor protein signaling pathway. [file 1755-8794-4-55-S2.PNG]

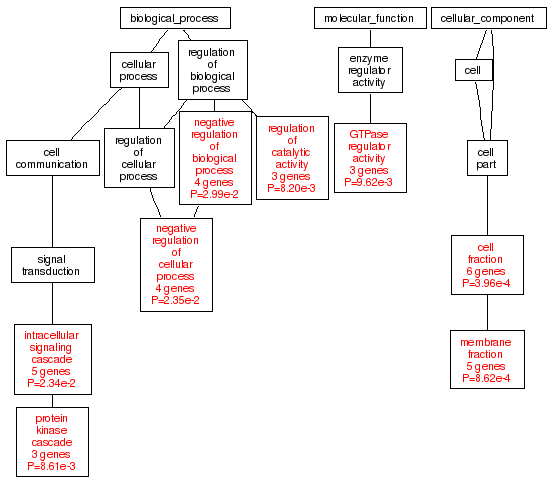

Supplement: Additional file 3 — Ontologies for the GSE5281 signature. Results of WebGestalt analysis of the gene set enrichment made for the GSE5281 signature in GO. The enriched ontologies are marked in red color. Similarly to the GSE1297 analysis, the GO subgraph associated to this signature is not large because of the scarce functionally characterizion of its genes. [file 1755-8794-4-55-S3.PNG]
